# French error type annotation for dictation: A platform with automatic error type annotation for French dictation exercises

**DOI:** 10.3389/fpsyg.2022.1075932

**Published:** 2023-01-12

**Authors:** Ying Qin, Yumeng Luo, Yuming Zhai

**Affiliations:** ^1^School of Information Science and Technology, Beijing Foreign Studies University, Beijing, China; ^2^Beijing Foreign Studies University (BFSU) Artificial Intelligence and Human Languages Lab, Beijing Foreign Studies University, Beijing, China

**Keywords:** artificial intelligence, AI program, language learning, language education, instant feedback, independent study

## Abstract

Dictation is considered an efficient exercise for testing the language proficiency of learners of French as a Foreign Language (FFL). However, the traditional teaching approach to dictation reduces the instructional feedback efficiency. To remedy this, this study adopts a design-based research approach and builds an automatic error type annotation platform for dictation practice named FRETA-D (French error type annotation for dictation) to pursue intelligent pedagogical feedback for both FFL teachers and students. FRETA-D can automatically identify error boundaries as well as classify the errors into fine-grained error types in learners’ dictation texts. FRETA-D features a dataset-independent classifier based on a framework with 25 main error types, which is generalized from French grammar rules and characteristics of frequent learner dictation errors. Five French teachers are invited to evaluate the appropriateness of automatically predicted error types of 147 randomly selected samples, and the acceptance rate reaches more than 85%. Automatic evaluation on 1,009 sentences by comparing with manually labeled references also shows promising results, reaching more than 85% consistency with human judgments. The accessibility of FRETA-D has also been confirmed by 50 Chinese undergraduate FFL learners with different professional backgrounds. FRETA-D facilitates conducting dynamic statistical analysis of learners’error types. And we share the same findings with previous studies that there exist causal links between the dictation errors and learners’ mastery of French phoneme and grapheme.

## 1. Introduction

Numerous studies have mushroomed on applying Artificial Intelligence (AI) to education, which opens new opportunities, potentials or challenges in education practices (Ouyang and Jiao, 2021). In the circumstance of foreign language teaching and learning, the technology-enhanced platforms which automatically evaluate EFL (English as a Foreign Language) students’ writings, translations, and dictations are increasingly appealing (Tang and Wu, 2017; Qin, 2019; Miao, 2021). Because these platforms can intelligently check the correctness of learners’ answers and return instructional feedback to them without the intervention of teachers.

However, most studies on AI-empowered language learning platform are centered on assisting English learning and teaching. This research domain needs to pay more attention to other languages. To fill this research gap, we developed an intelligent French dictation system in this study. In the Longman dictionary of Applied Linguistics (Richards and Schmidt, 2002), ‘dictation’ is defined as a technique used in both language teaching and language testing in which a passage is read aloud to students or test takers, with pauses during which they must try to write down what they have heard as accurately as possible. Dictation is a valuable technique for language learners to improve their listening and writing skills (Davis and Rinvolucri, 2002; Nation ISO, 2008; Sello and Michel, 2020). According to the comparative study of Rahimi (2008), dictation exercise can significantly improve the grammar, reading, vocabulary, and aural comprehension of learners. French language poses great challenges to learners by its phonological features intertwining with verb conjugation, gender/number agreement and orthography. As a result, dictation is viewed as a prized exercise in FFL (French as a Foreign Language) learning due to its holistic reflection of learners’ language proficiency, by simultaneously assessing learners’ listening comprehension and written production (Oual and Abadi, 2022). The traditional way of conducting paper-based dictation presents many shortcomings. It is not only time-consuming for the teacher to correct the learners’ copies, but also difficult to perform the systematic analysis of the learners’ errors. The learners moreover cannot receive instant feedback (Fairon and Simon, 2009; Beaufort and Roekhaut, 2011; Sanni, 2022).

We believe that an ideal intelligent dictation platform should be able to, upon learners’ submission, automatically detect the error boundaries, as well as classify the errors into fine-grained error types to provide pedagogical feedback. To achieve this research goal, we leveraged the recent research advances in the domain of automatic Error Type Annotation (ETA). Since each language possesses its own lexical and grammatical features, the error typology and ETA method cannot be directly applied across different languages. As a result, the existing ETA tools are mostly developed for specific languages. For example, ERRANT (Bryant et al., 2017) and SERRANT (Choshen et al., 2021) are for English, ARETA for Arabic (Belkebir and Habash, 2021), KAGAS for Korean (Yoon et al., 2022), and others for Portuguese (del Río and Mendes, 2018) and Czech (Rosen, 2017), etc. These tools are basically used for two purposes: (1) realizing automatic ETA in learner corpora, to reduce manual annotation effort, and provide more detailed linguistic information; and (2) evaluating more precisely the output of grammatical error correction systems (Bryant and Ng, 2015; Felice and Briscoe, 2015; Choshen et al., 2020). The automatic ETA is generally performed in learner essays or transcribed spoken texts. As far as we know, no previous tool has been developed to annotate French dictation errors, which could involve not only grammatical ones, but also phonetic-related ones. The latter needs our special treatment in developing our ETA tool. In particular, this study adapted the error typology and classification algorithm of ERRANT (Bryant et al., 2017), a toolkit developed to annotate English grammatical error types and whose general framework is the most similar to ours.

Previous studies have conducted manual error analysis in French dictation, which provide guidance in terms of error typology and error analysis for this study. Žugelj (2013) constructed a corpus of 112 French L2 dictation copies of Slovenian undergraduate students, based on which the author proposed an error typology and analyzed the causes of each error type. Cao and Wang (2014) collected 234 French L2 dictation copies of Chinese undergraduate students during their third year of French major study. The analysis showed that their errors were centered around the basic language knowledge and the overall discourse comprehension. The reading speed also had a major impact on their dictation performance. Escoubas-Benveniste and Di Domenico (2017) conducted an analysis on 214 French L2 dictation copies of Italian undergraduate students, to discover their verbal morphology acquisition. As for the regular verbs, students generated 62% of spelling errors on inflection morpheme. Qualitative analysis of some verbal contexts showed systematic spelling errors at various linguistic levels.

This study departs from a design-based approach (Sandoval and Bell, 2004) to design, develop and apply an intelligent dictation platform named FRETA-D, which is devoted to FFL teaching and learning. It serves as a teaching-promoting platform for Chinese teachers teaching French and a smart partner for FFL learners.^1^ Learners’ dictation texts are automatically saved and processed on the platform. Each error in the texts is annotated with a clear boundary and a fine-grained error type.

The significance of the study is reflected in the following aspects: (1) building up a fine-grained error typology specifically for French dictation exercises, covering both grammatical and phonetic errors; (2) developing a rule-based classifier to automatically annotate French dictation error types; and (3) freeing teachers from the burdens of correcting learners’ dictation, and improving dictation practice efficiency for learners.

Through this design-based research, this paper attempts to address the following research questions:

1. How to automatically detect error boundaries and classify error types in learners’ French dictation?
2. What are users’ attitudes toward this dictation platform?
3. What are the frequent errors committed by Chinese learners in French dictation? And what might have been the cause?

## 2. Research method and procedures

### 2.1. Research method

The present study follows a design-based approach (Sandoval and Bell, 2004), which means in order to solve real-life educational problems, researchers continuously improve the design based on users’ feedback from practice in a real and natural context, until all flaws are eliminated and a maximally reliable and effective design is achieved. Following Easterday et al. (2014), design-based research processes consist of 6 iterative phases in which designers need to: focus on the problem, understand the problem, define goals, conceive the outline of a solution, build the solution, and test the solution.

In this study, we focus on the inconveniences presented in traditional paper-based dictation, and we understand users’ need for innovative transformation in their French learning process. Our goal is to build an AI technology-enhanced dictation platform to provide intelligent and instant pedagogical feedback, by automatically locating learners’ errors and classifying them into fine-grained error types.

The outline of building the FRETA-D platform is composed of four steps: (1) collecting and preprocessing learners’ dictation data; (2) establishing the error typology in French dictation exercise; (3) automatically detecting the error boundary; and (4) realizing the automatic grammatical and phonetic error classification. The detailed development procedures are presented in the “Procedures”. Finally, we invited teachers and learners to evaluate the platform and gathered their feedback.

### 2.2. Participants

Participants were enrolled in compulsory FFL courses at Beijing Foreign Studies University. Two classes (50 learners and two teachers) were selected based on convenience sampling. Class 1 was composed of 27 undergraduate students (22 females and 5 males) and class 2 of 23 undergraduate students (22 females and 1 male). They were in their sophomore year at the time of the study. Learners’ average age was 20. These learners were not majoring in foreign languages but international business, law, computer science, etc. They were all native speakers of Mandarin, with English as their first foreign language, and had been learning English for 10 to 13 years. They studied French from scratch as a second foreign language. Their French level corresponded approximately to CEFR A1 or A2.^2^ Their two French teachers were Chinese and one of them taught the two classes. These participants performed dictations after class on the platform, by following teachers’ weekly assignment or at their own pace.

### 2.3. Procedures

#### 2.3.1. Data collection and preprocessing

Due to the lack of an efficient French handwriting OCR (Optical Character Recognition) tool, the FRETA-D platform currently cannot recognize French text written on paper. Therefore, students are not allowed to upload their scanned or photographed manuscripts. Their answers must be input as computer-editable texts for subsequent processing.

For this reason, learners are required to input French texts by keyboard or Apple Pencil while they are practicing dictation on the platform. This text-entering method sometimes makes it easy for students to violate some writing rules, for example, adding or missing spaces before or after a punctuation, using improper forms of punctuation marks, etc. These editorial errors are often overlooked by teachers in students’ paper-based dictation copies. But some problems must be pointed out when learners type French with keyboard, because they might be real space-related or spelling mistakes. Therefore, we separate these kinds of problems into two situations and preprocess the collected students’ texts with corresponding methods (see details in Table 1). The arrow indicates the result of processing. In the first situation, we ignore the minor errors by preprocessing learners’ input; while in the second situation, when learners forget to put a space before certain punctuations (e.g., colon, question mark, exclamation point), no preprocessing is performed and the error is pinpointed.

**Table 1:** Dictation texts preprocessing concerning punctuations and spaces.

	Preprocessing methods	Instances
Situation 1	Forms of single quotation marks are not viewed as errors and unified into a standard one.	*aujourd’hui/aujourd´hui → aujourd’hui (today)*
	Comma without a space followed is edited by adding a space after the comma.	*ensuite,Lucy est venue → ensuite, Lucy est venue (then, Lucy came)*
Situation 2	If there is no space before a colon, a question mark or an exclamation point, it’s considered as an error and no processing is performed.	①*inconvénients: il y a…* (correct format: *inconvénients* : *il y a…*) *(inconvenience: there are…)* ②*Bonjour, Feny!* (correct format: *Bonjour, Feny* !) *(Hello, Feny!)* ③*Ça va bien?* (correct format: *Ça va bien ?*) *(How is it going?)*

In order to facilitate the subsequent error boundary detection, we also make fine-grained delimitations of some tokens during preprocessing, mainly involving the following two cases:

- Common tokenization: adding a space before a comma or period (e.g., ensuite, je suis allé → ensuite , je suis allé; les cheveux . → les cheveux .) (then, I went; the hair);
- Space addition: adding a space after a single quotation mark where there is elision, except for the word “aujourd’hui (today)” (e.g., s’est → s’ est; d’inscription → d’ inscription) (has been; of inscription).

After the elaborate data collection and preprocessing steps, we finally built up the learners’ dictation exercise corpus containing 1,009 sentences. Each sentence was paralleled with the standard answer as the reference.

#### 2.3.2. Error typology in French dictation exercises

According to the observation of Swanson and Yamangil (2012), most error types in learners’ texts are related to the part-of-speech (POS) of words. Bryant et al. (2017) also proposed a grammatical error type framework based on POS tags to annotate errors in learners’ English essays. They extended the error types by adding three coarse-grained types including missing, unnecessary, and replacement, according to whether a token should be inserted, deleted, or substituted. The spelling and word order errors, which are not related to POS, were also considered in their error framework.

To establish the French dictation error typology in our study, we took the typology established by Žugelj (2013) as a reference, and adapted the grammatical error framework proposed by Bryant et al. (2017). Table 2 summarizes the resulting typology with comparison to the work of Bryant et al. (2017). This typology contains all the types defined in the work of Žugelj (2013). Totally there are 25 error types (shared types plus added types), of which 19 types are borrowed from Bryant et al. (2017). The 19 types are further divided into three sub-types, including POS-related, morphology-related and other. Taking the particularity of French language and learners’ dictation texts into consideration, we also deleted 5 error types nonexistent in French from the framework of Bryant et al. (2017) and replenished 6 error types. The operation tiers consist of missing, unnecessary, and replacement if existing, each tier assigned with a specific tag for the automatic annotation.

**Table 2:** Twenty-five error types in French dictation exercises.

Type			Operation tier		
			Missing	Unnecessary	Replacement
19 types shared with the framework of Bryant et al. (2017)	POS	Adjective	M:ADJ	U:ADJ	R:ADJ
		Adverb	M:ADV	U:ADV	R:ADV
		Noun	M:NOUN	U:NOUN	R:NOUN
		Verb	M:VERB	U:VERB	R:VERB
		Pronoun	M:PRON	U:PRON	R:PRON
		Determiner	M:DET	U:DET	R:DET
		Conjunction	M:CONJ	U:CONJ	R:CONJ
		Punctuation	M:PUNCT	U:PUNCT	R:PUNCT
	Other	Contraction	–	–	R:CONTR
		Orthography	–	–	R:ORTH
		Spelling	–	–	R:SPELL
		Word order	–	–	R:WO
		Other	–	–	R:OTHER
	Morphology	Adjective form	–	–	R:ADJ:FORM
		Noun inflection	–	–	R:NOUN:INFL
		Noun number	–	–	R:NOUN:NUM
		Verb form	M:VERB:FORM	U:VERB:FORM	R:VERB:FORM
		Verb agreement	–	–	R:VERB:SVA
		Verb tense	M:VERB:TENSE	U:VERB:TENSE	R:VERB:TENSE
6 types added in our work	Grammar	Adposition	M:ADP	U:ADP	R:ADP
		Verb participle	–	–	R:VERB:PARTICIPLE
		Derivational suffixes	–	–	R:DERIV
		Negation	M:NEGATION	U:NEGATION	R:NEGATION
	Aural comprehension	Continuous words	M:CONTINUOUS	U:CONTINUOUS	R:CONTINUOUS
		Phonetic	–	–	R:PHONETIC
5 types deleted from the framework of Bryant et al. (2017)		Particle	M:PART	U:PART	R:PART
		Preposition	M:PREP	U:PREP	R:PREP
		Morphology	–	–	R:MORPH
		Noun possessive	M:NOUN:POSS	U:NOUN:POSS	R:NOUN:POSS
		Verb inflection	–	–	R:VERB:INFL

Different from essay writing, dictation exercise requires learners to replicate the reference text as faithfully as possible under listening stimuli. Therefore, six frequent error types related to dictation practice, further divided into grammar and aural comprehension sub-types, were added to the error framework. The following are the explanations of these error types.

- Adposition. It refers to the errors related to French preposition including missing, unnecessary, and replacement cases, belonging to the grammatical error (e.g., *elle parlera son rêve → elle parlera de son rêve*) (*she talked her dream → she talked about her dream*).
- Verb participle. In French, four common past participle forms share identical pronunciation (i.e., *é, és, ée, ées*), and the suffix changes with the corresponding subject, auxiliary verb or direct object. The complex agreement rule and non-corresponding between spelling and pronunciation often cause dictation errors among FFL beginner learners. Therefore, when there is an inappropriate gender/number agreement at the end of the words in past participle form, this error type will be assigned (e.g., *passé → passée*) (*past*).
- Derivational suffixes. This error type is assigned when the word stem is correct but the derivational suffix is wrong (e.g., *voyager → voyageur*) (*travel → traveler*).
- Negation. It refers to the missing, redundant, or wrong negation words (e.g., *ne, n’, *pas,* plus*) (*no, not, no longer*).
- Phonetic. Phonetic error results from the deviation of aural comprehension. FFL students might misunderstand the meaning and write down the wrong tokens with identical or similar pronunciation (e.g., *elle partira pour homme → elle partira pour Rome*) (*she will go to man → she will go to Rome*).
- Continuous words. On the dictation platform, to simulate the actual in-class dictation practicing environment, the audio is played only once (with the whole text being read four times), and the playing progress is not adjustable except for a pause. Therefore, learners might miss continuous words (i.e., a consecutive sequence of words, which is sometimes related to the difficulty of understanding liaison phenomena), or write multiple unnecessary/wrong words due to their memory limitation or aural comprehension error. This kind of error frequently occurs in learners’ dictation texts (e.g., *ils sont un peu le santé → ils sont en bonne santé; Thomas est rue → Thomas est vite apparu*) (*they are a little the health → they are in good health; Thomas is street → Thomas appeared quickly*).

#### 2.3.3. Error boundary detection

In our error type annotation work, detecting the error boundaries is an important step to ensure the accuracy of locating errors and the following error classification. The task of error boundary detection is to mark the span of different tokens in the dictation text compared to the standard answer, which is fundamentally an alignment problem (Bryant et al., 2017).

Swanson and Yamangil (2012) made the first attempt at automatic error boundary detection by simply using the Levenshtein distance to align two parallel sentences. Levenshtein distance between two sentences is defined as the minimum number of token edits including insertion, deletion, or substitution. However, the standard Levenshtein distance only aligns individual tokens and does not allow for token transposition. In other words, it cannot identify word order errors such as [A B → B A] and instead identifies it as [A → Ø], [B → B] and [Ø → A], which does not meet the requirements of most error typologies. To address this problem, Swanson and Yamangil (2012) merged all adjacent non-matches, and Xue and Hwa (2014) subsequently improved their work by training a maximum entropy classifier to avoid unnecessary merging between two errors. Brill and Moore (2000) proposed Damerau-Levenshtein distance, which was an extension of Levenshtein distance and was also capable of identifying word order errors.

Felice et al. (2016) applied Damerau-Levenshtein distance to this task with two significant modifications: (1) incorporating linguistic information such as lemma and part-of-speech tags into the alignment; and (2) implementing a rule-based merging function to decide whether two or more single-token edits (i.e., aligned error-correction pairs) should be merged into a multi-token edit (e.g., [sub → subway] + [way → Ø] = [sub way → subway]). As a result, this method achieved the best performance on all datasets they used. These approaches of improvement were proved to produce more accurate, natural and human-like alignments, and were finally adopted in ERRANT by Bryant et al. (2017).

**Figure 1:**
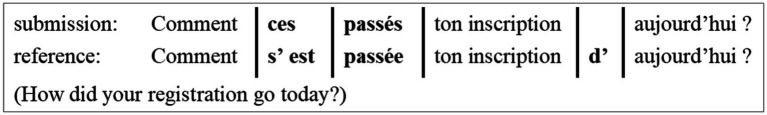
Illustration of error boundary detection (the black vertical lines indicate the boundaries of errors).

In this study, we applied the alignment algorithm of Felice et al. (2016) to our submission-reference parallel sentences, without modifying the linguistic information and merging rules in their algorithm. An example of detected error boundaries is illustrated in Figure 1. We consider it as a pretty ideal alignment because “*ces*” (*these*) and “*s’est*” (*has been*) have similar phonetic transcription and they are merged into the same edit, which is very beneficial to the subsequent error type annotation work.

#### 2.3.4. Error classification

A pipeline of error classification is built to assign error type tags after the boundary detection step as described above. The parallel dictation text and reference are first processed by a natural language processing toolkit named spaCy^3^ (version 2.3.7), to generate rich linguistic information including POS, lemmatization, stemming, and dependency analysis. The sentence pairs are then input to the grammatical error classifier to judge whether there exist grammar errors. Next, the phonetic error classifier further judges whether there exist phonetic errors and marks them up. That is to say, an error may be assigned more than one error tag if it belongs to multiple types.

##### 2.3.4.1. Grammatical error classification

**Figure 2:**
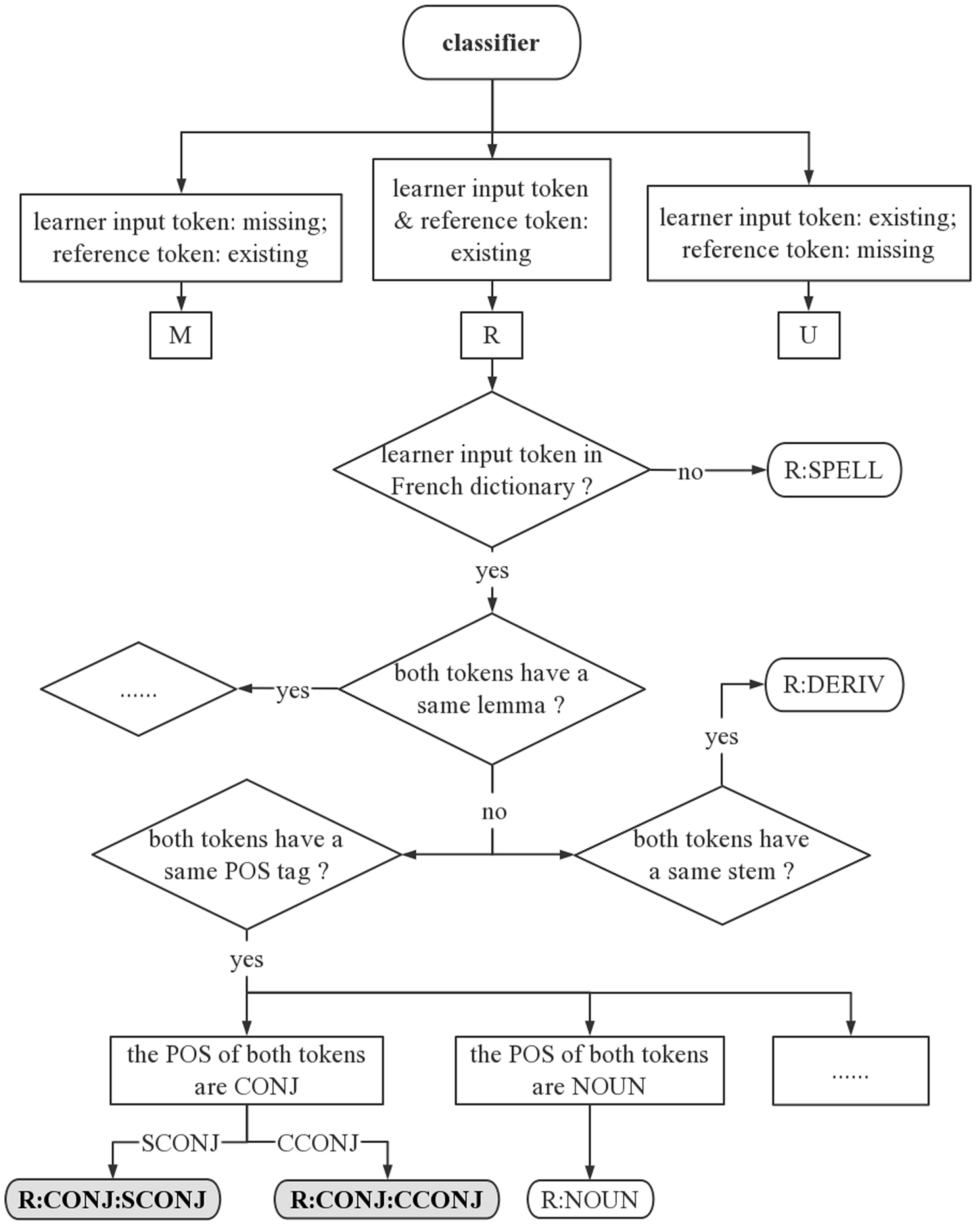
Examples of grammatical error classification. R:SPELL, R:DERIV, R:NOUN, R:CONJ:SCONJ, and R:CONJ:CCONJ are classified using logic tree.

We implemented a rule-based logic tree to classify the grammatical errors in French dictation texts. Multiple linguistic information is required to apply these rules, including POS, lemmatization, stemming, and dependency relations. For example, to detect the error of conjunction, we search all subordinate conjunction (with POS tag SCONJ) or coordinating conjunction (with POS tag CCONJ) in the reference. If *que, comme, et, ou (that, since, and, or)* etc. is missing in the corresponding edit of the student’s submission, it will be annotated with the error type *M: CONJ*. If these words should be replaced by others, it will be classified as error *R: CONJ*. In total, we apply 20 grammar rules to recognize grammatical errors. Figure 2 shows detailed annotation examples of several error types in the rule-based logic tree.

##### 2.3.4.2. Phonetic error classification

Phonetic errors are challenging for FFL learners. Since there exist many homonyms in French (e.g., “*vert, ver, vers, verre*”) (*green, bug, towards, glass*), the listening stimuli are not sufficient for learners to choose the correct word during the dictation and they need to resort to the context to make the right decision. The phonemes nonexistent in Chinese also pose challenges for French aural comprehension, such as distinguishing voiced and voiceless consonants, distinguishing similar vowels (e.g., [ø] vs. [ɔ]), recognizing liaison phenomena, memorizing silent word-final consonants, etc.

In order to detect the phonetic errors in the exercises, we have to first obtain accurate phonetic transcriptions of each word. We use the python library Phonemizer^4^ (Bernard and Titeux, 2021) to transcribe words on both sides of an edit into International Phonetic Alphabet (IPA), and calculate their similarity.

Rule-based methods are applied to recognize the phonetic errors after IPA transcription. We summarize seven rules with examples as shown in Table 3 (the arrow indicates the correct form). The rules describe the conditions to be satisfied before classifying the edit as a phonetic error, and these conditions could coexist in the same edit.

**Table 3:** Rules and examples for phonetic error classification.

Number	Rule description	Examples
1	Erroneous and correct tokens are both monosyllabic (with elision or not) and they contain the same or similar vowels or consonants	*un → en, ou → au, est → et (a → at, or → at the, is → and)*
2	Erroneous and correct tokens are both polysyllabic and the pronunciation only differs in one or several pairs of voiced vs. voiceless consonants	*cadeau → gâteau, dans → t’en (gift → cake, in → to you)*
3	Erroneous and correct tokens are both polysyllabic and they share identical pronunciation	*apprendre → à prendre (learn → to take)*
4	Learners wrongly take the pronunciation of the ending sound in liaison phenomena for a consonant at the beginning of the next word	*est d’âgée → est âgée (is … years old)* *est d’au → est au (is at the)*
5	Mishearing numbers	*dix-huit → dix-sept (eighteen → seventeen)*
6	Failing to recognize the word and spelling it in the wrong form by only relying on the listening stimuli	*y sera → essaiera (there will be → will try)* *en soleil → ensoleillé (in the sun → sunny)*
7	Due to silent word endings, omitting or writing redundant word-final “s” or “e,” or misspelling the verb conjugation suffix	*veux → veut (want: first/s-person conjugation → third-person conjugation)* *il → ils (he → they)*

#### 2.3.5. M^2^ format and feedback

**Figure 3:**
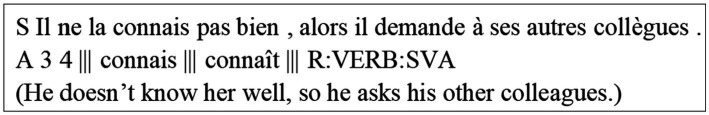
Error annotation in M^2^ output format.

To increase the readability and make it more convenient to check the automatic markup information during the development, we use the M^2^ output format (Dahlmeier and Ng, 2012) with slight modifications to present error annotation results. An example is shown in Figure 3. The learner’s dictation answer is shown in the first line and the error is marked underneath.

Each dictation sentence has a capitalized S at the beginning of its line. The format of error output consists of: *A start-index end-index|||wrong token |||correct token|||error type tag*. In this example, the erroneous word “*connais*” and reference word “*connaît*” share the same pronunciation, and they are, respectively, the second-person and third-person conjugated forms of the verb “*connaître (know)*.” Hence this is classified as a subject-verb disagreement error. This error annotation format is easy to be utilized by the platform to perform further statistical analysis.

**Figure 4:**
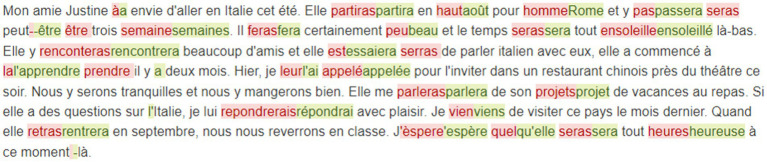
The feedback interface of the dictation platform.

During the dictation exercise, the platform automatically checks learners’ submission against the reference, marking the errors in red and showing the reference at the same time in green. Figure 4 demonstrates the feedback interface of the dictation platform for learners. Currently we are working on improving the classification accuracy of the classifier. When it meets our expectations, we will add instant error type annotation on the platform.

## 3. Evaluation and results

We conducted four experiments to evaluate the platform’s performance, which are summarized as follows.

In terms of overall evaluation of the error annotation, we conducted manual and automatic evaluation:

1. We invited five Chinese teachers teaching French (all from Beijing Foreign Studies University) to manually evaluate the error annotation results of a small-scale dataset (147 edits) and gathered their feedback (see “Manual evaluation of error annotation”).
2. We conducted an automatic evaluation of error annotation on a larger dataset (2,092 edits) (see “Automatic evaluation of error annotation”).

To investigate the strength and weakness of the classifier on different error types, we conducted the diagnosis evaluation (see “Diagnosis evaluation”). Finally, to evaluate the accessibility of the platform (see “The analysis of the platform”), we invited 50 Chinese FFL learners and their two teachers, all from Beijing Foreign Studies University, to participate in this study.

### 3.1. Overall evaluation

#### 3.1.1. Manual evaluation of error annotation

We first carried out a small-scale manual evaluation by inviting 5 Chinese teachers teaching French to rate the performance of error annotation in dictation exercises. To conduct the evaluation, we randomly selected 147 preprocessed edits with error types automatically annotated by the classifier pipelines. Then the teachers were told to evaluate each error type as *Good*, *Acceptable,* or *Bad*. *Good* means the predicted type is the most appropriate for the given edit. *Acceptable* means the predicted type is appropriate but probably not optimum. While *Bad* means the predicted type is not appropriate for the edit. The teachers were also invited to put forward the reasons and suggestions when they did not choose *Good* or when they did not agree with the classification results. The result of the manual evaluation of 5 teachers on 147 samples is shown in Table 4.

**Table 4:** Result of manual evaluation of predicted error types on 147 samples.

Rater	Good	Acceptable	Bad
1	81.70%	9.50%	8.80%
2	67.60%	2.80%	29.60%
3	94.60%	2.00%	3.40%
4	72.60%	9.60%	17.80%
5	87.20%	1.30%	11.50%
Average	80.74%	5.04%	14.22%

The error annotations rated as *Good* or *Acceptable* are summed up to 85.78%. It is fairly good and is applicable in French dictation practice.

We further examined the effect of personal subjectivity of raters on the evaluation. We calculated the inner consistency among the five raters using Fleiss’s Kappa (Cardillo, 2007). The Kappa coefficient of agreement among the raters was only 0.21. The rather low agreement among human raters reflected the great difficulty of automatic error type annotation. By analyzing the teachers’ suggestions, we found that some raters tended to choose *Acceptable* when they thought that an annotated error type was not fully appropriate, while others preferred to choose *Bad* when they did not fully agree with the classification result or the error typology, which accounted for the higher *Bad* percentage.

**Figure 5:**
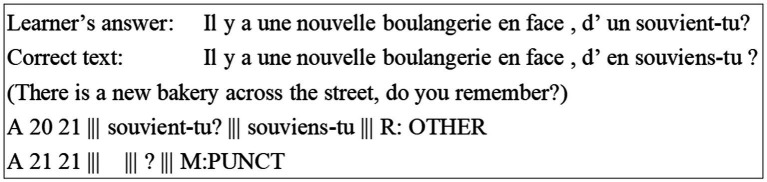
An example of noise introduced by automatic edit extraction.

We also looked into the annotation results which were evaluated as *Acceptable* or *Bad* and summarized them into three cases:

- Some rules generated too coarse-grained error types. The raters thought that these errors should be annotated into other types. For example, some raters considered that the error types *Continuous words* and *Other* are too general to indicate error information;
- Some annotation errors originated from the language preprocessing pipeline, including POS tagging and dependency parsing. For example, [*veux → veut*] (*want: first/s-person conjugation → third-person conjugation*) might be viewed as a VERB error rather than a more fine-grained VERB: SVA error due to the wrong POS tagging result;
- The automatic edit extraction may bring noise, e.g., [*souvient-tu? → souviens-tu*] and [→?] (See Figure 5) are two automatically extracted edits but such alignment results are not helpful to identify the error types VERB: SVA (*souvient → souviens*) and ORTH (missing a space before a question mark).

#### 3.1.2. Automatic evaluation of error annotation

To further evaluate the performance of FRETA-D on learners’ dictation exercises of a larger scale, we applied it to the corpus containing 1,009 sentences. Each sentence was paralleled with a standard answer as a reference and each error in the sentence was manually annotated with gold standard error types. In this corpus, each sentence may have one or more edits (e.g., *ces→ce*) (*these→this*). There were a total of 2,092 edits to annotate. It’s worth mentioning that the error type framework with the same classification criteria was applied to both the automatic annotation by FRETA-D and manual annotation. The automatic comparison results between the types predicted by FRETA-D and the reference types are shown in Table 5. Among the 2,092 errors, both grammatical/spelling and phonetic errors obtained consistency rates higher than 85% (respectively 1,798 and 1,812 predicted types consistent with the reference types). It was close to the acceptance rate evaluated by the 5 human raters (85.78%).

**Table 5:** Automatic evaluation results on 1,009 sentences.

	Consistent	Inconsistent	Total
Grammatical/Spelling errors	1,798 (85.95%)	294 (14.05%)	2,092 (100%)
Phonetic errors	1,812 (86.70%)	280 (13.30%)	2,092 (100%)

### 3.2. Diagnosis evaluation

To show the detailed performance of the classifier on different error types, following Bryant et al. (2017), we carried out the diagnosis evaluation as shown in Table 6.

**Table 6:** Annotation performance on each error type.

Type	Precision	Recall	F1-score	Type	Precision	Recall	F1-score
ADJ	0.66	0.91	0.77	NOUN:NUM	0.98	0.81	0.89
ADJ:FORM	0.89	0.70	0.78	ORTH	1.00	1.00	1.00
ADP	1.00	1.00	1.00	OTHER	0.75	0.86	0.80
ADV	0.82	0.82	0.82	PRON	0.94	1.00	0.97
CONJ	0.92	0.92	0.92	PUNCT	0.97	0.95	0.96
CONTINUOUS	0.90	0.95	0.93	SPELL	1.00	0.91	0.95
CONTR	0.83	0.96	0.89	VERB	0.65	0.73	0.69
DERIV	0.47	0.80	0.59	VERB:FORM	0.91	0.73	0.81
DET	0.95	0.87	0.91	VERB: PARTICIPLE	0.89	0.69	0.77
NEGATION	1.00	1.00	1.00	VERB:SVA	0.75	0.33	0.46
NOUN	0.88	0.86	0.87	VERB:TENSE	0.35	0.39	0.37
NOUN:INFL	0.00	0.00	0.00	WO	1.00	1.00	1.00
PHONETIC	0.85	0.86	0.86				

The shade values 1.00 mean the highest value of precision, recall and F1-score.

The following conclusions are drawn from Table 6.

1. In total, the F1 scores of 88% types (22 out of 25) were higher than 0.5, revealing that our automatic annotation is quantitatively as good as manual annotation to some extent.
2. The classifier performed perfectly on some types including ADP, NEGATION, ORTH and WO, and 6 error types (CONJ, CONTINUOUS, DET, PRON, etc.) obtain F1 higher than 0.9. Only three types were poorly annotated with F1 less than 0.5.
3. Half of our added types reached the Recall of more than 0.9 (ADP and NEGATION with score of 1, and CONTINUOUS with 0.95), and none of the deleted types occurred in the automatic annotation results, which indicates success of our modification to the original framework of ERRANT (Bryant et al., 2017).
4. It is worth noting that the type NOUN: INFL (wrong gender agreement or gender & number agreement of nouns) was scored 0, which means that the classifier cannot correctly recognize this type.

Two possible causes of the poorly annotated error types are: (1) the natural language processing toolkit spaCy provides incorrect linguistic information for types like NOUN: INFL, DERIV, or VERB: PARTICIPLE; and (2) the classification logic is not comprehensive enough and needs further optimization.

### 3.3. The analysis of the platform

We investigated the 50 learners’ attitudes toward the accessibility of the platform and whether they could adapt themselves to it. Questionnaires were distributed to collect learners’ feedback and 31 valid copies were retrieved (18 from Class 1 and 13 from Class 2). The questionnaire contained 12 open-ended questions and the responses amounted to 6,479 Chinese characters in total. We conducted a thematic analysis on the survey data through inductive coding with the software NVivo (Bazeley and Jackson, 2013). The steps included preparing and organizing data, reading through data, data coding, theme mining, result presentation and data interpretation (Creswell, 2013).

Most respondents preferred handwriting to typing whenever it’s possible. Because they think writing is more fluent and quicker than typing hence making them feel more at ease. Almost all the respondents agreed that they were adapted to entering text by keyboard or Apple Pencil on the platform.

Through qualitative analysis of learners’ opinions, the three most prominent advantages of the platform include: (1) immediate correction upon submission; (2) the possibility to redo the exercise; and (3) flexibility compared to the “class-based” approach in terms of time and space. Learners also indicated that they can hear the audio more clearly and they feel less nervous when practicing online. It was also reported that the platform saved precious teaching time in class and gave learners free access to more dictation materials.

In contrast, the four most prominent disadvantages include: (1) challenge posed by typing rather than writing; (2) absence of collective learning and feeling of being challenged in a real-time evaluation situation; (3) slackness due to the lack of supervision; and (4) impossibility to ask questions as in class.

All the respondents agreed that the platform contributed to improve their dictation skills and the main reasons are as follows: (1) the platform allows for more practice after class, which guarantees a steady input and output ratio in language learning; (2) more practice improves their familiarity with French pronunciation, grammar, and vocabulary; and (3) the immediate feedback helps to quickly identify their flaws.

## 4. Discussion and future work

### 4.1. Analysis of learners’ errors

The platform also facilitates conducting the dynamic statistical analysis of learners’ error types for teachers. Of the 470 samples chosen from dictations of 22 FFL learners (sum = 470, mean = 21, std. = 17), we sorted out 820 error-correction pairs and found that most of the errors are lexico-syntactically related in Chinese FFL learners’ dictations (i.e., with context, learners could have solicited either lexical or syntactical model in their language knowledge to exclude the erroneous form). In other words, in the circumstance that learners have much time to examine their output after the audio is played, a crucial factor that causes a poorly performed dictation is the non-mastery of vocabulary and grammar. This finding supports the rationality of our approach of setting a series of grammar-related error types, by incorporating various linguistic information to describe Chinese FFL learners’ dictation errors.

There are causal links between the dictation errors and the learners’ mastery of French phoneme and grapheme. Firstly, the recognizability of words is reduced by the abundance of open syllables in word formation or the monosyllabicity of functional words; secondly, French has many minimal phoneme pairs containing inexistent phonemes in Chinese (e.g., [œ] and [ø]; or voiced consonants like [b], [d] and [g] that are not differentiated in Chinese). These two aspects were also reported by Cao and Wang (2014), where the errors caused by “confusing similar pronunciation” accounted for 32.84% in their corpus. Thirdly, French grammatical features such as verb endings and gender-number agreement are often silent or homonymic. The resulting grapheme-phoneme non-correspondence brings great challenges for beginning learners. This is consistent with the analysis of Žugelj (2013).

At the same time, this study shares the findings of Escoubas-Benveniste and Di Domenico (2017) that contextual prosodic effects can alter word segmentation and influence the writing process. The errors could derive from an erroneous processing of the binding consonant. When learners wrongly interpret a consonant as a stable phoneme rather than a contextual prosodic phenomenon, an unnecessary phoneme will be written (e.g., *est d’au → est au*) (*is at the*).

### 4.2. Conclusion and future work

We built up a platform of automatic error type annotation for French dictation exercises, serving as an independent learning and self-learning partner for learners. Errors in learners’ dictation texts are related not only to grammatical but also to phonetic difficulties. However, most available tools used for grammatical error type annotation are designed for English. This study first proposes the error type framework for French dictation practice considering grammatical and phonetic problems in learners’ copies. We implemented an automatic error type annotation pipeline consisting of two stages: error boundary detection and error type classification. The manual and automatic evaluation results were satisfying. The users also confirmed the advantages of the platform.

As far as we know, there is fewer study on error type annotation of French dictation in China and abroad. This platform can find its way into computer-assisted French teaching and provide an intelligent learning experience for learners. To further improve the performance of this platform, we have the following goals and challenges:

- Refining and adding edit merging rules in the phase of error boundary detection;
- Exploring more tools that are sustainable, accurate, and effective at identifying French liaison in phonetic transcription and improving the phonetic error classification;
- Tackling the mistakes caused by natural language processing tools, such as errors from POS tagging, and preventing the error propagation in the pipeline;
- Optimizing the defective error classification rules by exploring detailed and subtle ones.

When the classifier’s performance meets our expectations, we’ll provide automatic error type annotation on the platform. Users’ attitudes toward the effectiveness of this new function will be qualitatively analyzed through questionnaires and open-ended interviews.

In the future, the platform could be combined with more data analysis technology to quantitatively analyze the distribution of learners’ dictation error types, and provide visual and intuitive feedback for FFL learners and teachers. We will further consider each learner’s difficulties and provide personalized learning materials or create enhancing exercises for them, making it an advanced platform for autonomous and active learning.

## Data availability statement

The raw data supporting the conclusions of this article will be made available by the authors, without undue reservation.

## Author contributions

All authors listed have made a substantial, direct, and intellectual contribution to the work and approved it for publication.

## Funding

This work was supported by the Fundamental Research Funds for the Central Universities “Database construction for French textbooks and dictation exercises” (grant number 2022JS002), and the National Emerging Humanity and Social Sciences Research and Reform Practice Project “Developing Undergraduate Programs in Foreign Languages and Artificial Intelligence” (grant number 2021060009).

## Conflict of interest

The reviewer ZL declared a shared affiliation, with no collaboration, with one of the authors to the handling editor at the time of the review.

## Publisher’s note

All claims expressed in this article are solely those of the authors and do not necessarily represent those of their affiliated organizations, or those of the publisher, the editors and the reviewers. Any product that may be evaluated in this article, or claim that may be made by its manufacturer, is not guaranteed or endorsed by the publisher.

## Footnotes

^1^Since dictation could be practiced at any level with appropriate materials, the platform is suitable for FFL learners of all levels.

^2^This simply corresponded to the level of the textbook that they used.

^3^ https://github.com/explosion/spacy-models/releases//tag/fr_core_news_md-2.3.0

^4^ https://github.com/bootphon/phonemizer
